# Ammonia-Oxidizing Archaea Are More Resistant Than Denitrifiers to Seasonal Precipitation Changes in an Acidic Subtropical Forest Soil

**DOI:** 10.3389/fmicb.2017.01384

**Published:** 2017-07-24

**Authors:** Jie Chen, Yanxia Nie, Wei Liu, Zhengfeng Wang, Weijun Shen

**Affiliations:** ^1^Center for Ecological and Environmental Sciences, South China Botanical Garden, Chinese Academy of Sciences Guangzhou, China; ^2^College of Life Science, University of Chinese Academy of Sciences Beijing, China; ^3^Department of Soil Science of Temperate Ecosystems, University of Göttingen Göttingen, Germany

**Keywords:** ammonia-oxidizing archaea, denitrifiers, nitrogen transformation, precipitation change, resistance index, subtropical forest

## Abstract

Seasonal precipitation changes are increasingly severe in subtropical areas. However, the responses of soil nitrogen (N) cycle and its associated functional microorganisms to such precipitation changes remain unclear. In this study, two projected precipitation patterns were manipulated: intensifying the dry-season drought (DD) and extending the dry-season duration (ED) but increasing the wet-season storms following the DD and ED treatment period. The effects of these two contrasting precipitation patterns on soil net N transformation rates and functional gene abundances were quantitatively assessed through a resistance index. Results showed that the resistance index of functional microbial abundance (-0.03 ± 0.08) was much lower than that of the net N transformation rate (0.55 ± 0.02) throughout the experiment, indicating that microbial abundance was more responsive to precipitation changes compared with the N transformation rate. Spring drought under the ED treatment significantly increased the abundances of both nitrifying (*amoA*) and denitrifying genes (*nirK, nirS*, and *nosZ*), while changes in these gene abundances overlapped largely with control treatment during droughts in the dry season. Interestingly, the resistance index of the ammonia-oxidizing archaea (AOA) *amoA* abundance was significantly higher than that of the denitrifying gene abundances, suggesting that AOA were more resistant to the precipitation changes. This was attributed to the stronger environmental adaptability and higher resource utilization efficiency of the AOA community, as indicated by the lack of correlations between AOA gene abundance and environmental factors [i.e., soil water content, ammonium (NH_4_^+^) and dissolved organic carbon concentrations] during the experiment.

## Introduction

Changes in seasonal precipitation distribution are increasingly severe in tropical and subtropical areas, with intensified droughts in winter and spring while more storms in summer (Cleveland et al., 2010; Wu et al., 2010; Sui et al., 2012; Zhang et al., 2012). However, the consequences of belowground processes and biological communities caused by these precipitation changes have been under studied (Austin et al., 2004; Allison and Treseder, 2008; Cleveland et al., 2010; Wu et al., 2011). The soil nitrogen (N) cycle and its associated properties are vital indicators of soil fertility that determines plant productivity and ecosystem functions (Yandjian et al., 2011). The by-products of nitrate (NO_3_^-^) and nitrous oxide (N_2_O) during the N cycle are important sources of N loss, soil pollution, and greenhouse gasses (Fang et al., 2009; Isobe et al., 2012). A better understanding of the effects of predicted seasonal precipitation changes on soil N transformations is necessary to evaluate the responses of regional ecosystem functions. However, previous studies regarding the responses of soil N transformation rates to altered precipitation patterns focused mostly on arid and semi-arid areas (Beier et al., 2012). Further studies about the responses of soil N transformation processes and functional microbes to seasonal precipitation changes in tropical and subtropical areas are urgently needed (Beier et al., 2012; Liu et al., 2016).

Soil microorganisms play an important role in controlling soil biogeochemical cycles, soil nutrient availabilities, and plant productivity; therefore, they are considered as the fundamental part of terrestrial ecosystems (Harris, 2003, 2009; van Dijk et al., 2009; Banning et al., 2011). It has been shown widely that soil microorganisms are sensitive to environmental changes, such as soil moisture and organic carbon (C) and N contents, which are easily influenced by extreme drought and precipitation events (Reichmann et al., 2013; Nielsen and Ball, 2015). Changes in specific functional microbial groups are used increasingly as indicators of changes in ecosystem functions, as they show strong correlations with soil nutrient cycles in various environments (Rasche et al., 2011; Petersen et al., 2012). However, few studies have investigated the responses of N-cycle-associated microorganisms to predicted precipitation changes (Fuchslueger et al., 2014). To our knowledge, whether functional microorganisms and N transformation rates respond similarly to precipitation changes, as well as to what extent soil microorganisms are indicative of N cycle responses, remains debatable (Bell et al., 2014; Fuchslueger et al., 2014).

Ammonium-oxidizer (AO) communities carrying the *amoA* gene are the most important microbial groups in the N cycle, as they drive the rate-limiting and central step of nitrification: ammonium oxidation (Levy-Booth et al., 2014). Denitrifying microorganisms harboring the *nirK, nirS*, and *nosZ* genes are also important in the N cycle, as they catalyze the nitrite-reducing and nitrous-oxide-reducing processes of denitrification (Morales et al., 2010; Rasche et al., 2011; Petersen et al., 2012). Both nitrifiers and denitrifiers are sensitive to changes in soil moisture and ammonium (NH_4_^+^) and dissolved organic carbon (DOC) concentrations (Wankel et al., 2011; Levy-Booth et al., 2014). Specifically, AO communities are characterized by a slow regeneration rate and mixotrophic growth, which confer starvation tolerance and extreme environmental adaptability (Levy-Booth et al., 2014; Fujitani et al., 2015), especially for archaeal ammonium-oxidizers (AOA). The AOA community has been reported in environments with low ammonium contents (Lam et al., 2007; Hatzenpichler et al., 2008), low pH (Gubry-Rangin et al., 2011) or extreme temperatures (Hatzenpichler et al., 2008; Chen et al., 2016). Contrastingly, denitrifiers are heterotrophic and anaerobic microorganisms that need high substrate availability and soil water content (SWC) (Szukics et al., 2010; Levy-Booth et al., 2014). The distinct environmental preferences between nitrifiers and denitrifiers may result in different responses of these microbial groups to altered environments under precipitation changes.

The responses of target variables to disturbances can be quantified by a resistance index as proposed by Orwin and Wardle (2004) to evaluate the amount of change in response variables caused by a disturbance. Recently, this index has been used successfully to evaluate the abilities of microorganisms and soil physiochemical properties to resist disturbances (Orwin et al., 2006; Bouskill et al., 2013; Delgado-Baquerizo et al., 2014; Thion and Prosser, 2014). For example, the resistance index of a soil microbial community structure was found to be a good indicator of the stability of soil respiration and other soil functions in response to a drying/rewetting cycle (Orwin et al., 2016). As suggested by plant-based ecology theory, fast-growing microorganisms that tend to apply the *r*-strategy may have greater abundances and lower abilities to adapt to environmental changes, and less resistance to disturbances. Contrastingly, slow-growing microorganisms that tend to apply the *K*-strategy may have high abilities to remain stable after disturbances (Orwin et al., 2006; de Vries and Shade, 2013). Nevertheless, little information is known about the differences in resistance abilities between nitrifiers and denitrifiers, and whether their resistance abilities are associated with their life strategies remains poorly understood.

In this study, seasonal precipitation changes were regarded as disturbances. The responses of soil N transformation rates, and nitrifying and denitrifying microorganisms were evaluated and compared via the Orwin-Wardle resistance index. Two contrasting patterns of seasonal precipitation changes were manipulated in a subtropical forest in southern China for 2 years to mimic on-going regional precipitation changes. One pattern incorporated a drier dry season and a wetter wet season (hereafter the DD treatment) and another pattern incorporated an extended dry season and the wetter wet season (hereafter the ED treatment). This experiment aimed to (1) investigate the effects of different precipitation changes (i.e., the DD and ED treatments) on soil N transformation rates and functional gene (*amoA, nirK, nirS*, and *nosZ*) abundance, (2) quantify and compare the responses of soil N transformation rates and functional gene abundance, and (3) evaluate the resistance of the nitrifiers and denitrifiers to environmental changes caused by the altered precipitation patterns.

## Materials and Methods

### Site Description

The study was conducted in the Heshan National Ecosystem Observation and Research Station, Chinese Academy of Sciences (112° 54′ E, 22° 41′ N), Heshan, Guangdong Province, southern China. The study forest is a 35-year-old evergreen broadleaved mixed-species forest, and the climate type is south subtropical monsoon, with an average annual temperature of 21.7°C and an average annual precipitation of 1,700 mm (Zhao et al., 2017). This region is characterized by a typical wet season (April through September) in which it receives 80% of its annual rainfall, and a dry season (October through March) in which it receives only 20% of its annual rainfall (Chen et al., 2017). The annual rainfall of the two study years were within the normal range, although the 1st year (2013, 2,094 mm) was relatively wetter than the 2nd year (2014, 1,551 mm). Soil in this forest is Oxisol that developed from sandstone. The forest has 11 tree species and 26 shrub species, and detailed information of the vegetation features are presented in Supplementary Figure S1.

### Experimental Design

A field precipitation-manipulating experiment with two precipitation treatments (the DD and ED treatments) was conducted for 2 years from October 2012 to September 2014. The DD treatment was excluding 67% of throughfall from October to March and adding the equivalent excluded rainwater back during April to September, namely drier dry-season and wetter wet-season treatment (DD). The ED treatment was excluding 67% of throughfall from April to May and adding the equivalent excluded rainwater back during June to September, which was defined as extending dry-season and wetter wet-season treatment (ED). The adoption of these precipitation treatments was mainly based historical and projected precipitation changes in the subtropical region of China. After analyzing the precipitation data from 1950 to 2009 in subtropical China, Zhou et al. (2011) found that the number of rainy days decreased significantly during the dry season (from October to March), while large rain events (50–100 mm day^-1^) increased significantly during the wet season (from April to September), which was the main basis for our DD treatment. Additionally, spring drought also occurred frequently in this subtropical area (Zhang et al., 2012), which provided the basis for our design of the ED treatment. The observed increases in large storms during the wet season are also projected to occur in many other regions of the world (IPCC, 2013).

To implement these precipitation treatments, twelve 12 m × 12 m experimental plots with an adjacent distance ≥ 2 m were assigned randomly to four replicates of each treatment and a control. To reduce the potential for lateral water movement and surface run-off from surrounding areas, a 60–80-cm deep trench was excavated, and a 1-m-high polyvinyl chloride (PVC) segregation board was imbedded around the perimeter of each plot. The precipitation-manipulating facilities consisted of supporting structures, rainfall exclusion sheets, and water addition subsystems, which were constructed in the DD and ED treatment plots (Supplementary Figure S2). Sixteen galvanized steel pipes (2.5–3-m length × 10-cm diameter) were welded together with eight horizontal stainless steel frames (12-m length) at the top and then vertically fixed in concrete bases that were imbedded in the soil to a depth of 60 cm to form the supporting structures. In each treatment plot, 8–12 rainout sheets (length, 12 m; width, 50–100 cm), with a total area occupying 67% of the plot, were fixed in two stainless steel frames and hanged on the supporting system with steel hooks. The rainout sheets were made from polyethylene plastic with >90% light transmission, which will not affect the temperature or the level of irradiation from sunlight (Reichmann et al., 2013). For the DD treatment plots, the sheets were opened to exclude 67% of the throughfall during the dry season (October 1 through March 31), but they were folded so that they did not exclude throughfall during the wet season (April 1 through September 30). For the ED treatment plots, the sheets were opened to exclude 67% of the throughfall during the spring season (April 1 through May 31), but they were folded so that they did not exclude throughfall during the late wet season (June 1 through September 30). A water pump connected to the plots with PVC and rubber pipes served as the water addition facility. The water was pumped from a nearby pond and transported through the PVC and rubber pipes, and it was distributed evenly by 25 sprinklers in each plot. Prior to the experiments, chemical properties of the pond water and rainwater were checked several times. The results showed that the nutrient contents, including N and organic C levels, were slightly higher in the rainwater, but the pH was similar (rainwater, 6.42; pond water, 6.19). This ensured that we did not add nutrients while adding water. The amount of water added during the wet season was calculated as a product of the above-canopy dry-season rainfall, the throughfall ratio, and the throughfall exclusion ratio (0.67). The above-canopy rainfall was obtained from a standard meteorological station (Davis, Vaisala, Finland), and the throughfall ratio was 0.86, which was obtained from eight rain gauges (TB4MM, Techno Solutions, Beijing, China) that were installed randomly in eight plots. The exact amount of precipitation excluded and the date for the water addition are described in Supplementary Table S1.

### Soil Sampling and Analysis

Soil samples were collected at the end of each treatment. For the DD treatment, sampling was conducted after the throughfall reductions (March 27, 2013, and March 26, 2014) and water additions (August 28, 2013, and August 289, 2014). For the ED treatment, soil samples were collected on May 28, 2013, and May 30, 2014, after the throughfall reductions, and on September 20, 2013, and September 27, 2014, after the water additions. Six topsoil (0 to 10 cm) samples were collected randomly in each plot, with four samples located at the precipitation exclusion areas and another two samples were exposed to ambient precipitation. The six samples were pooled and mixed homogeneously to form one composite sample for further analyses. After removing litterfall and stones, the soil samples were stored immediately in an ice box and then transported back to the laboratory. The soil samples were sieved through a 2-mm mesh, and divided into two parts, with one part stored at 4°C for soil physicochemical analysis and the other stored at -20°C for microbial analysis. All samples were analyzed within 2 weeks after sampling.

Soil physicochemical properties were measured using methods described by Liu et al. (1996) and Chen et al. (2015). Briefly, the SWC was detected by drying the soil sample at 105°C for 24 h in a cylindrical stainless steel container of known weight. Total organic carbon, total N, and total phosphorus, NH_4_^+^ and NO_3_^-^ concentrations were measured using the K_2_Cr2O7 oxidation, semi-micro Kjeldahl, ascorbic acid colorimetric, indophenol blue colorimetry, and copperized cadmium reduction methods, respectively (Liu et al., 1996). Soil pH testing was performed at a soil to water ratio of 1:2.5 using a pH meter (Denver Instrument UB-7 pH, Denver Instrument, Arvada, CO, United States). Soil microbial biomass carbon (MBC) and DOC was measured using the fumigation extraction method (Vance et al., 1987). Briefly, 10 g of fresh soil was placed into a glass beaker and fumigated with chloroform in a vacuum glass dryer for 24 h in the dark. Meanwhile, another 10 g of fresh soil was extracted with 0.5 M K_2_SO_4_. After fumigation, the soil was also extracted with 0.5 M K_2_SO_4_, and the extracted liquids from both the fumigated and non-fumigated soils were measured for the DOC content on a total organic C analysis instrument (TOC-VCSH, Shimadzu, Kyoto Japan). Then, the MBC was calculated as the difference of the DOC concentration between the fumigated and non-fumigated samples modified by the extraction factor of 0.45 (Vance et al., 1987). The DOC concentrations in the non-fumigated samples were considered as the original soil DOC concentrations.

Net N mineralization and nitrification rates were measured using the resin-core method through an *in situ* field soil incubation (Reichmann et al., 2013). Near the six soil samples in each plot, another six topsoil cores were incubated for 1 month in six PVC pipes (diameter, 4.6 cm; length, 12 cm) with an ion-exchange resin bag in the bottom to measure the leached inorganic N. The total concentrations of NH_4_^+^ and NO_3_^-^ after incubation were measured from the resin and soil cores. Then, the net N mineralization rate was calculated as the NH_4_^+^ and NO_3_^-^ concentrations after the incubation minus the initial NH_4_^+^ and NO_3_^-^ contents obtained from the six soil samples 1 month previously. The net nitrification rate was calculated as the NO_3_^-^ concentration after the incubation minus the initial NO_3_^-^ content (Reichmann et al., 2013).

Soil total DNA extraction and purification were performed using the HiPure Soil DNA Mini Kit (Magen, Guangzhou, China). Measurements of the abundances of archaeal *amoA* and denitrifying genes (i.e., *nirK, nirS*, and *nosZ*) were performed on an ABI 7500 thermocycler system (Applied Biosystems, Foster City, CA, United States) with absolute real-time polymerase chain reaction (PCR) protocol as described by Chen et al. (2015). The real-time PCRs were performed in a 20-μl volume containing 12.5 μl of SYBR Premix Ex Taq (TaKaRa Biotechnology, Japan), 1 μl of each primer (10 mmol/L), 2 μl of DNA template (10 ng), and 1 μl of dimethyl sulfoxide. A three-step amplification method was used with the following conditions: 95°C for 30 s, followed by 40 cycles of 5 s at 95°C, 34 s annealing, and 1 min at 72°C. The primers and annealing temperatures used in the real-time PCR protocals were *amoA* 1F/*amoA* 2R and 55 °C for bacterial *amoA* (Petersen et al., 2012; Levy-Booth et al., 2014), *CrenamoA* 23f/*CrenamoA* 616r and 53°C for archaeal *amoA* (Tourna et al., 2008; Levy-Booth et al., 2014), F560-589/ R906-935 and 65°C for *nirK* (Chenier et al., 2003), *nirS* 1F/*nirS* 3R and 65°C for *nirS* (Braker et al., 1998; Levy-Booth and Winder, 2010), and *nosZ* 2F/*nosZ* 2R and 65°C for *nosZ* (Henry et al., 2006). Gene copy numbers in the samples were calculated directly from standard curves (Henry et al., 2006; Isobe et al., 2012). To construct the standard curves, the target gene PCR products were cloned into the pMD20-T vector (TaKaRa, Dalian, China) to form the cloning fragments and then transformed into the *Escherichia coli* JM109 strain. The recombinant *E. coli* JM109 strains were inoculated in Luria–Bertani broth containing ampicillin and incubated overnight at 37 °C. Then, the recombinant plasmids carrying the target genes were extracted using the HiPure Plasmid Mini Kit (Magen, Guangzhou, China) and quantified on a NanoDrop 2000 spectrophotometer (Thermo Fisher Scientific Inc., Waltham, MA, United States). DNA copy numbers of the extracted plasmids were calculated from the plasmid sizes, concentrations, and average base pair molecular weights. Then, a standard curve was generated from a 10-fold serial dilution (10^3^–10^8^ copies per μl) of the plasmids.

### Calculation of the Resistance Index

The stabilities of functional gene abundance, soil physicochemical properties, and net N transformation rates, in terms of their resistance to precipitation changes, were quantified by resistance indices as described by Orwin and Wardle (2004) and Delgado-Baquerizo et al. (2014) with following equation:

$$R_{s}\;=\;1\;-\;(2\times (|C_{0}\;-\;S_{0}|)/(C_{0}\;+\;|C_{0}\;-\;S_{0}|))$$

where *C*_0_ and *S*_0_ are the values from the control and treatments, respectively, at the end of each treatment period. For example, values from the control and DD treatment were used to calculate the resistance index for the DD treatment, and values from the control and ED treatment were used to calculate the resistance index for the ED treatment. The value of the resistance index (*R*_S_) is bounded between -1 and 1. If the index value reaches 1, this indicates that the treatment did not cause any change in the response variable. If the index value is 0 or negative, this means that there is a 100% change or greater than 100% change in the response variable in the treatment compared with that in the control (Orwin et al., 2006).

### Statistical Analysis

One-way analysis of variance with multiple comparisons was used to test the differences of the resistance index among different functional genes. Two-way repeated-measures analysis of variance with sampling time as the repeated factor was used to examine the effects of precipitation changes (i.e., the DD or ED treatments) and sampling time on all measured parameters. Prior to the two-way repeated-measures analysis, Pillai’s trace from a multivariate test was used for within-subject tests when the assumption of multi-sample sphericity was not met. Independent sample *t*-tests were used to compare the observed values of each variable between the treatments and control, and the difference in the resistance index of each variable between the DD and ED treatments at each sampling point. Correlations between soil physicochemical properties and the resistance indices of functional gene abundances were tested using Spearman’s correlation analysis. All the parameters were explored for normality (Kolmogorov–Smirnov test) and homogeneity of variances (Levene’s test) prior to the analyses, and log-transformed if necessary. The statistical analyses described above were performed in SPSS for Windows v.16.0 (SPSS Inc., Chicago, IL, United States). All the data, including soil physicochemical properties, net N transformation rates, and functional microbial abundance, were subjected to principal component analyses (PCAs). The PCAs were performed on R 3.3.2 (R Core Team, 2012) with the package “FactoMineR.”

## Results

### Overview of Soil Physicochemical Properties

**FIGURE 1:**
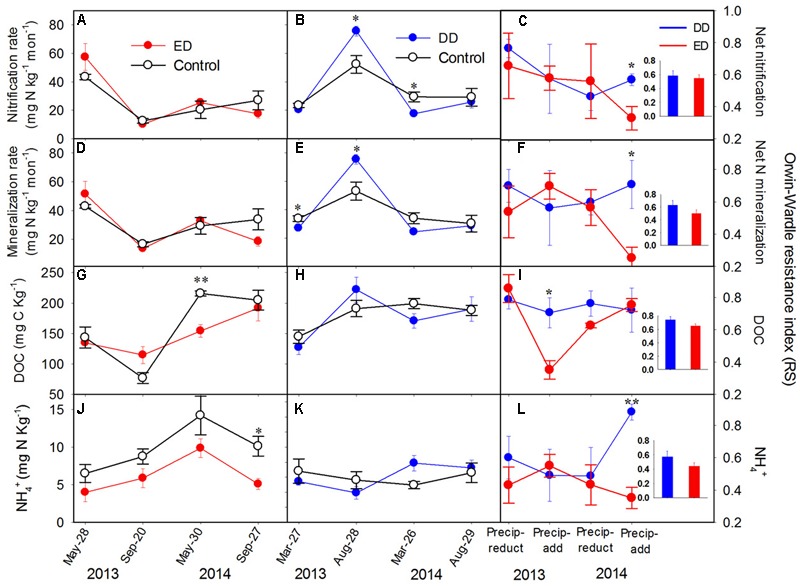
Dynamics of **(A–C)** net nitrification rate, **(D–F)** net N mineralization rate, **(G–I)** soil dissolved organic carbon (DOC) content, and **(J–L)** NH_4_^+^ concentrations in the control and treatment plots, and their resistance indices throughout the experiment (*n* = 4). The insert bar charts represent the average values of the resistance index in each treatment during the experiment. The *y*-axis of the middle and left panels is presented on the left side, and the *y*-axis of the right panel is presented on the right side. The significance of the differences between the treatments and control at each sampling point is presented as ^∗∗^*p* < 0.01, ^∗^*p* < 0.05. Abbreviations: DD, dryer dry season and wetter wet season; ED, extended dry season and wetter wet season; Precip-reduct, precipitation reduction; Precip-add, precipitation addition.

Vegetation features and the soil physicochemical properties showed no significant differences among the control, DD, and ED plots before the treatments (Supplementary Figure S1 and Table S2). Generally, each plot had 21 ± 3 individual trees with an average tree height of 7.9 ± 1.0 m, an average diameter at breast height (DBH) of 11 ± 1 cm, and an average crown width of 1.1 ± 0.1 m. There were about 88 ± 4 individual shrubs/herbs in each plot with an average values of height of 0.2 ± 0.03 m, an average diameter at basal area of 0.1 ± 0.01 cm, and an average crown width of 1.8 ± 0.8 cm. Regarding to the soil pH values (data not shown), there were no significant differences among the plots, with average values of 4.05 ± 0.08 and 3.78 ± 0.07 in the DD plots, 4.11 ± 0.1 and 3.94 ± 0.09 in the ED plots, and 4.11 ± 0.07 and 3.86 ± 0.10 in the control plots before and after the experiment, respectively. During the experiments, the ED treatment had significant effects on MBC (*p* < 0.01) and NH_4_^+^ (*p* < 0.01), and the interaction between ED treatment and sampling time (ED × time) showed strong effects on MBC, DOC, NO_3_^-^, and net nitrification and N mineralization rates (*p* = 0.075, **Figures 1A,D,G,J**). The DD treatment only had significantly negative effects on SWC, but the interaction between DD treatment and sampling time (DD × time) had pronounced effects on most soil properties (*p* = 0.076; i.e., DOC, SWC, NO_3_^-^, and net nitrification and N mineralization rates, **Figures 1B,E,H,K**).

### Resistance of Functional Gene Abundance and Soil Physicochemical Properties to Precipitation Changes

**FIGURE 2:**
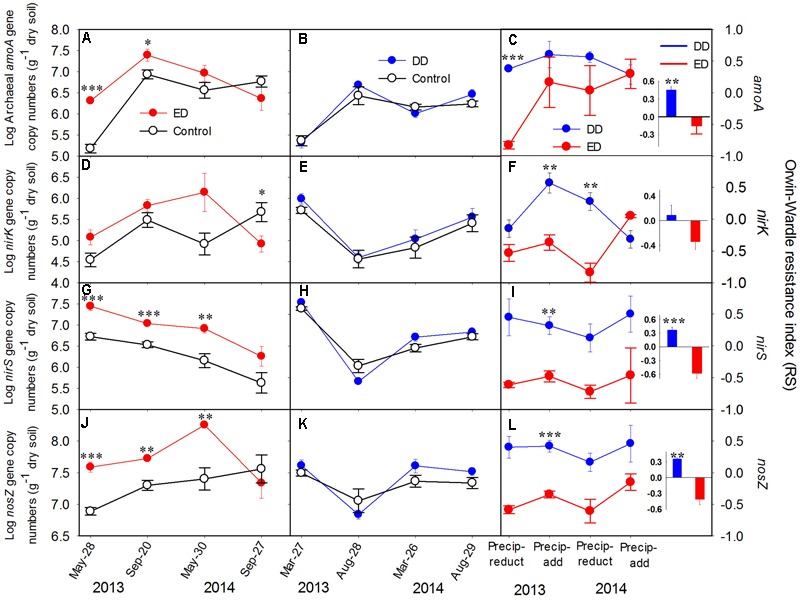
Log-transformed copy numbers of **(A–C)** archaeal *amoA*, **(D–F)** *nirK*, **(G–I)** *nirS* and **(J–L)** *nosZ* genes in the control and treatment plots, and their resistance indices throughout the experiment (*n* = 4). The insert bar charts represent the average values of the resistance index in each treatment during the experiment. The *y*-axis of the middle and left panels is presented on the left side, and the *y*-axis of the right panel is presented on the right side. The significance of the difference between the treatments and control at each sampling point is presented as ^∗∗∗^*p* < 0.001, ^∗∗^*p* < 0.01, ^∗^*p* < 0.05. Abbreviations: DD, dryer dry season and wetter wet season; ED, extending dry season and wetter wet season; Precip-reduct, precipitation reduction; Precip-add, precipitation addition.

No amplification of the bacterial *amoA* gene was detected during our experiments, which was mainly due to the low soil pH in the studied areas. This is in agreement with previous studies reporting low ammonia-oxidizing bacteria abundance in acidic soils (Gubry-Rangin et al., 2011; Isobe et al., 2012). Thus, only the AOA community was examined in the following analyses. During the 2-year experiment, the abundances of archaeal *amoA, nirS*, and *nosZ* genes were affected significantly by the ED treatment (*p* < 0.05), while *nirK* gene abundance was altered significantly by the effects of the interactions between ED and time (*p* < 0.01, **Figures 2A,D,G,J**). All the functional gene abundances showed an increasing trend throughout the ED treatment, while decreasing to the control level after the water addition in 2014 (**Figures 2A,D,G,J**). Overall, the annual average abundances of the *amoA, nirS*, and *nosZ* genes were increased significantly by the ED treatment. Contrastingly, the DD treatment did not cause any statistically significant changes in any of the functional gene abundances (**Figures 2B,E,H,K**). The ED treatment had stronger effects on these gene abundances than the DD treatment, which is in accordance with the pronounced decreased resistance indices of the functional gene abundances in the ED treatment, which reached low, negative values during 2 years of treatment (**Figures 2C,F,I,L**). The negative values of the resistance indices indicated that more than 100% of the changes in these functional gene abundances were caused by the ED treatment. However, the relatively higher, positive resistance indices from the DD treatment suggested that it caused fewer changes in these functional gene abundances (see the insert charts in **Figure 2**). Similarly, the soil net nitrification rate and N mineralization rate, MBC, and the DOC, NH_4_^+^, and NO_3_^-^ concentrations were more strongly affected by the ED treatment than the DD treatment, and they exhibited lower resistance indices in the ED treatment (**Figure 1** and Supplementary Figure S3). However, the resistance indices of the soil physicochemical properties were higher than those of the functional gene abundances in both the ED and DD treatments (see the insert charts in **Figures 1, 2**, and Supplementary Figure S3), suggesting that the precipitation changes had a greater effect on the functional gene abundances than the soil physicochemical properties.

Multiple comparisons of the resistance indices of different functional genes revealed that the archaeal *amoA* gene abundance had a greater resistance index than those of the denitrifying genes at most sampling points, except for the precipitation reduction in 2013 during both the dry and spring seasons, and the water addition in 2014 during the wet season (**Table 1**). The resistance indices of the denitrifying gene abundances became negative after most treatment points during the experiment. Contrastingly, negative resistance index values of *amoA* gene abundance were only observed after the precipitation reduction of DD treatment in 2013 (**Table 1**). The correlations between soil properties and the resistance indices of functional gene abundances were tested by Spearman’s correlations (**Table 2**). During the 2 years of disturbance caused by precipitation changes, the resistance indices of the denitrifying gene abundances correlated strongly and positively with soil DOC concentration, while they correlated negatively with SWC and soil NO_3_^-^ concentration (*p* = 0.09, **Table 2**). In contrast, the resistance index of archaeal *amoA* gene abundance did not show any significant relationship with any of the measured soil physicochemical properties (**Table 2**).

**Table 1:** Values of the resistance index of functional gene abundances in the DD and ED treatments (means and standard errors, *n* = 4) in 2013 and 2014, respectively.

	Treatment		Sig. *p*-value	Archaeal *amoA*	*nirK*	*nirS*	*nosZ*
2013	DD	Precip-reduct	0.11	0.38(0.07)^a^	-0.15(0.24)^b^	0.45(0.50)^a^	0.40(0.30)^ab^
		Precip-add	0.51	0.60(0.36)^a^	0.57(0.27)^a^	0.32(0.28)^a^	0.42(0.19)^a^
	ED	Precip-reduct	0.07	-0.83(0.12)^a^	-0.53(0.23)^b^	-0.61(0.08)^ab^	-0.59(0.13)^b^
		Precip-add	0.22	0.17(0.69)^a^	-0.37(0.21)^a^	-0.48(0.15)^a^	-0.34(0.10)^a^
2014	DD	Precip-reduct	0.19	0.57(0.17)^a^	0.28(0.28)^ab^	0.12(0.38)^b^	0.17(0.29)^b^
		Precip-add	0.11	0.29(0.27)^ab^	-0.32(0.24)^b^	0.50(0.49)^a^	0.46(0.50)^a^
	ED	Precip-reduct	0.11	0.04(0.68)^a^	-0.84(0.26)^b^	-0.72(0.18)^ab^	-0.61(0.37)^ab^
		Precip-add	0.23	0.30(0.40)^a^	0.05(0.04)^a^	-0.46(0.75)^a^	-0.15(0.26)^a^

*The differences of the resistance indices among the four genes were assessed by a one-way analysis of variance. Different lowercase letters indicate significant differences at *p* < 0.05. Abbreviations: DD, dryer dry season and wetter wet season; ED, extended dry season and wetter wet season; Precip-reduct, precipitation reduction; Precip-add, precipitation addition.*

**Table 2:** Spearman’s correlation (*r*) between the resistance indices of microbial attributes and soil physicochemical properties with significant levels at ^∗∗^*p* < 0.01, ^∗^*p* < 0.05.

Resistance index	DOC	SWC	NO_3_^-^	NH_4_^+^	NNR	NMR
Archaeal *amoA*	0.27	-0.19	-0.004	0.28	-0.13	-0.02
*nirK*	0.51^∗∗^	-0.49^∗^	-0.03	-0.16	-0.05	0.04
*nirS*	0.23	-0.36	-0.34	-0.12	0.05	0.001
*nosZ*	0.35	-0.37^∗^	-0.27	-0.15	0.06	0.002
MBC	0.12	-0.38^∗^	-0.03	-0.25	0.00	-0.05

*DOC, dissolve organic carbon (mg C kg^-*1*^); MBC, microbial biomass carbon (mg C kg^-*1*^); NNR, net nitrification rate (mg N kg^-*1*^ month^-*1*^); NMR, net N mineralization rate (mg N kg^-*1*^ month^-*1*^).*

**FIGURE 3:**
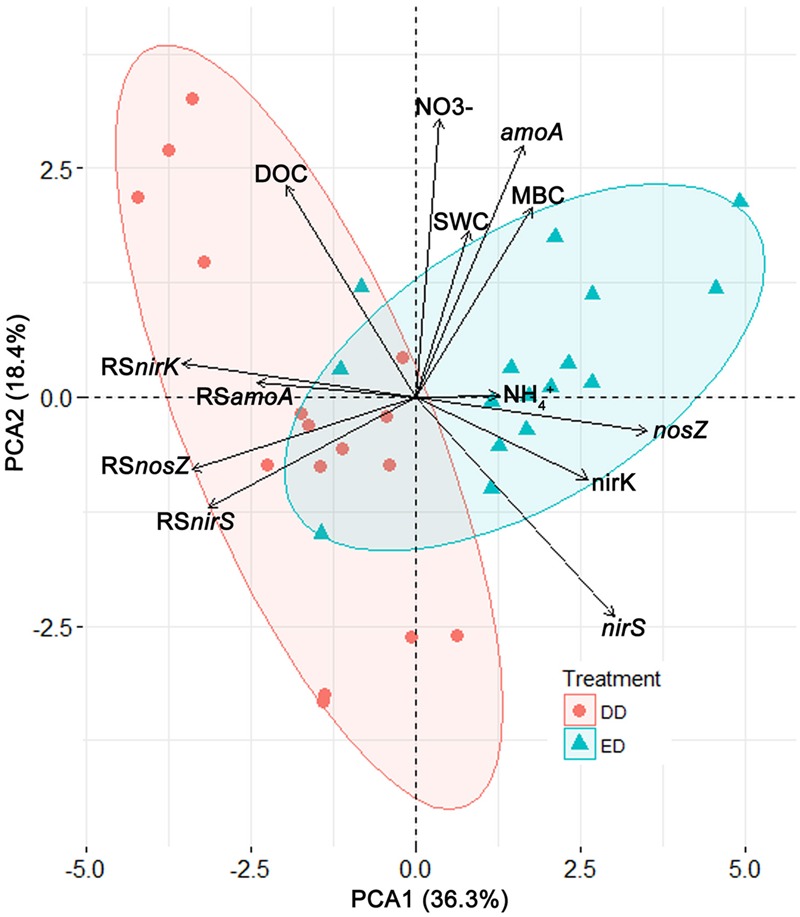
The biplot of the principal component analysis results represented by the first two principle components (PC1 and PC2). Deviation ellipses were drawn to show the distribution of the points from the different treatments. Vectors display the variables contributing to the two principle components. Abbreviations: SWC, soil water content; MBC, microbial biomass carbon; DOC, dissolved organic carbon; RS*amoA*, RS*nirK*, RS*nirS*, RS*nosZ*, resistance indices of the abundances of the *amoA, nirK, nirS, nosZ* gene, respectively.

To characterize and illustrate the effects of the DD and ED treatments, a PCA was conducted based on all the data from the DD and ED plots. The first two principle components (PC1 and PC2) were retrieved and used in a biplot (**Figure 3**). PC1 explained 36.3% of the total variance, and it correlated significantly and positively with the ED treatment, MBC concentration, and functional gene abundances, while it correlated negatively with the DD treatment, DOC concentration, and the resistance indices of functional gene abundances. PC2 explained 18.4% of the total variance, and it correlated positively with SWC, MBC, DOC, and NO_3_^-^ concentrations, and *amoA* abundance, while it correlated negatively with *nirS* abundance, and was not affected by treatment. The ED and DD treatments were separated significantly along PC1 (*R^2^* = 0.53, *p* < 0.001). More specifically, the ED treatment was characterized by high MBC concentrations and functional gene abundances, which contrasted with the DD treatment that exhibited high DOC concentrations and resistance indices of functional gene abundances. These patterns further support our results showing significantly higher resistance of functional gene abundances in the DD treatment than the ED treatment (**Figures 2C,F,I,L**).

## Discussion

### Functional Gene Abundance and Soil Net N Transformations Respond Differently to Precipitation Changes

In this study, the responses of soil N transformation-related properties and functional gene (*amoA, nirK, nirS*, and *nosZ*) abundances to two contrasting seasonal precipitation patterns were investigated. Except for *nirK*, all the functional gene abundances were affected significantly by the ED treatment, with pronounced increases in the spring drought. However, the seasonal patterns of these functional gene abundances in the DD treatment overlapped largely with the natural variations. These results indicate that drought has stronger effects on nitrifying and denitrifying microorganisms during the spring than during the dry season. Because the soil was originally dry and suffered from a substrate deficiency during the dry season, compared with the spring (see the control in **Figures 1G,H,J,K** and Supplementary Figure S3), the effects of drought on microorganisms and N transformation rates during the dry season might be obscured by the natural seasonal variations (**Figures 1, 2**). Contrastingly, spring drought could have greater impacts on these soil properties, because plants and soil microorganisms grow rapidly with increasing precipitation and temperature during spring (Wang et al., 2016). Here, we found that spring drought significantly increased functional gene abundances and the MBC concentration, but it decreased the NH_4_^+^ concentration (**Figures 1J, 2** and Supplementary Figure S3A). This was unexpected, because reduced precipitation during the spring usually has negative effects on soil microbial activities (Allison and Treseder, 2008). This contrasting result may be attributed to the combined effects of changes in SWC and substrate availability. More specifically, previous studies demonstrated that substrate limitations were the major inhibitors of nitrifiers when the soil water potential was greater than 20.6 MPa (Stark and Firestone, 1995). Although few studies have investigated the substrate limitation thresholds of denitrifiers under various moisture conditions, evidence has shown significant relationships among soil moisture, N substrates, and the abundance and structure of denitrifying communities (Szukics et al., 2010; Levy-Booth et al., 2014). The average SWC from April to May was higher than 30% during this study, which might be too wet for nitrifiers and denitrifiers to survive in the presence of limited substrates. Thus, soil with a lower SWC caused by a precipitation reduction during spring could relieve the substrate limitation and provide better conditions for these functional microorganisms. This hypothesis is further supported by the significant decrease of NH_4_^+^ concentration, as NH_4_^+^ might be used intensively by the increasing numbers of nitrifying and denitrifying microorganisms (**Figure 2**). However, the decreased SWC during the dry season did not affect these functional gene abundances, which might be due to the suppressive effect of lower temperatures during the dry season than in spring. Our results address the importance of seasonal concerns when conducting precipitation-manipulating experiments. Although the subtropical forests are facing extreme climate events, such as droughts and floods, the spring drought will have greater impacts on the soil ecosystem. Moreover, the functional gene abundances were increased significantly by the spring drought (**Figures 2A,D,G,J**), which might result in an open N cycle and more soil N loss via leaching and denitrification after a sustained spring drought.

However, the responses of the net N mineralization and nitrification rates were not in line with the responses of the functional gene abundances, as much greater changes were observed in the latter (**Figures 1, 2**). This is consistent with other studies showing sensitive responses of soil microbial communities to precipitation change disturbances (Bell et al., 2014; Fuchslueger et al., 2014; Orwin et al., 2016). Unlike these previous studies, we used the resistance index to quantify the responses, which made it possible to compare the stabilities of microorganisms and soil processes after precipitation changes. As a result, the net N mineralization and nitrification rates showed much greater resistance than the microbial abundances (**Figures 1C,F, 2C,F,I,L**), suggesting smaller changes in the N transformation rates. One possible reason for these results is the short observation time after treatment, which might only catch the responses of microbial communities, as the responses of soil net nutrient transformations to a disturbance often lag. The soil net N transformation rates are not influenced merely by microbial activities, but by plant absorption, which respond slowly to disturbances. It has been shown that the responses of roots to temporal changes in soil NH_4_^+^ and NO_3_^-^ concentrations are slower than those of microorganisms, which results in a temporal niche differentiation between roots and microorganisms (Kuzyakov and Xu, 2013). Plant absorption contributes substantially to soil NH_4_^+^ and NO_3_^-^ concentrations after incubation, which may control changes in soil net N transformations. Therefore, the slow responses of plant uptake to environmental changes could result in slower responses of net soil N transformation rates compared with microbial responses. A longer observation time might be necessary to test the responses of soil net N transformation rates to precipitation changes. Another possible reason for these results is the functional redundancy of soil microorganisms, as the soil N transformation rates and nutrient availabilities could also be controlled by some fungal communities that are more resistant to disturbances than the bacteria measured in this study (Griffiths et al., 2004; Wardwell et al., 2008; Chaer et al., 2009). Consequently, significant shifts in functional archaeal and bacterial communities could only induce slight changes in associated soil biogeochemical cycles. It was suggested previously that changes in the soil microbial community could serve as indicators of ecosystem responses to disturbances, as microorganisms are not followers but facilitators (Harris, 2009). This study demonstrated that the responses of functional gene abundance were more sensitive to precipitation changes than the soil net N transformation rates, which further confirms the important roles played by soil microorganisms when assessing the responses of ecosystems to climate change.

### Different Resistances of Nitrifiers and Denitrifiers to Precipitation Changes

This study demonstrated that AOA abundance was more resistant to drought or storms than denitrifier abundance (**Figures 2F,G,I,L**), indicating that the precipitation changes had greater effects on denitrifiers. Although the *amoA* gene abundance correlated positively with the DOC concentration and the SWC in the PCA biplot, the changes in the SWC and the DOC concentration caused by our treatments may not be sufficient to induce significant alterations in *amoA* abundance, as AOA can live in environments with a low SWC and DOC concentrations (Zhalnina et al., 2012). The resistance index of AOA abundance did not correlate with the soil physicochemical properties (**Figure 3** and **Table 2**), which suggests that the AOA community is strongly tolerant of environmental changes. This agrees with previous studies reporting a wide ecological niche of the AOA community (Erguder et al., 2009). However, the AOA community was less abundant than denitrifiers throughout the experiment (**Figures 2A,B,H,J,K**). These results suggest that the AOA community resists disturbance mainly through enhancing its environmental adaptability, rather than increasing its regeneration rate and biomass accumulation (Schimel et al., 2007).

According to different life-history strategies, microorganisms with high growth rates and low resource use efficiencies are *K*-strategists, while microorganisms with low growth rates and high resources use efficiencies are *r*-strategists (de Vries and Shade, 2013). Furthermore, microorganisms using the *K*-strategy are more resistant to disturbances than those using the *r*-strategy (Bapiri et al., 2010; de Vries et al., 2012). In this study, we found that the AOA community had a greater ability to resist precipitation changes than denitrifiers, and that the *amoA* gene was less abundant than the denitrifying genes (**Figure 2**). According to the features of different life history strategists, the AOA community is more likely to be a *K*-strategist, while the denitrifiers are more likely to be *r*-strategists. Although many of the bacteria conducting specialized or narrow functions in soil have been proven to be *r*-strategists (Schimel et al., 2007), the AOA community may be different, as archaea have specific cellular structures and physiological features compared with bacteria (Baumeister and Lembcke, 1992; Nelson et al., 2012). However, further investigations of the life-history strategies of AOA and denitrifiers are needed. In addition to simply growing and dying, microorganisms may respond to disturbances by changing their physiological state or by dispersing from nearby communities (Shade et al., 2012; de Vries and Shade, 2013). Here, the AOA community might have a greater ability to enter dormancy or to improve its resource use efficiency to maintain the community size under drought and substrate limitation stresses resulting from precipitation changes, as AOA have an extreme environmental tolerance (Chen et al., 2016). Furthermore, the dispersal of denitrifiers might be more limited by the SWC than the AOA community, as denitrification is performed under anaerobic environments (Levy-Booth et al., 2014). The higher stability of AOA abundance in response to precipitation changes, compared with that of denitrifiers, suggests that such disturbances may alter the composition of soil functional microbial groups and, consequently, associated soil N transformation processes.

## Conclusion

This study investigated the responses of soil net N mineralization and nitrification rates, and nitrifying and denitrifying gene abundances to seasonal precipitation changes in a subtropical forest in southern China. The throughfall reduction in spring had greater impacts on both the net N transformation rates and functional gene abundances than during the drought in the dry season. However, the responses of functional gene abundances to the spring drought were much more sensitive than those of the net N transformation rates, as indicated by higher resistance indices of the net N transformation rates. Although the abundances of both AOA community and denitrifiers increased during the experiment, the AOA community exhibited a significantly higher resistance index than the denitrifiers, which might be associated with a greater resource use efficiency and stronger environmental adaptability of the AOA community. These results suggest that the responses of functional microorganisms could be more effective in indicating the responses of soil ecosystems to precipitation changes. Moreover, the nitrifiers and denitrifiers showed different abilities to resist precipitation changes, which might depend on their life-history strategies.

## Author Contributions

JC conducted the experiment, analyzed the data, and wrote the initial draft of the manuscript. WS conceived the study. All authors contributed to revising the manuscript.

## Conflict of Interest Statement

The authors declare that the research was conducted in the absence of any commercial or financial relationships that could be construed as a potential conflict of interest.
