# Supplementary material for: Ammonia-Oxidizing Archaea Are More Resistant Than Denitrifiers to Seasonal Precipitation Changes in an Acidic Subtropical Forest Soil
Source: Front Microbiol. 2017 Jul 24;8:1384. doi: 10.3389/fmicb.2017.01384 (PMC5522861; doi:10.3389/fmicb.2017.01384)
Supplement: Supplementary file 1 [file Data_Sheet_1.docx]

***Supplementary Material***

**Ammonia-oxidizing archaea** **are more resistant than denitrifiers to seasonal precipitation changes** **in an acidic subtropical forest soil**

**Running tittle:** Microbial resistance to precipitation change

# Author: **Jie Chen ^1,2,3^, Yanxia Nie^1^, Wei Liu ^1^, Zhengfeng Wang ^1^, Weijun Shen ^1*^**

**^1^ Center for Ecological and Environmental Sciences, South China Botanical Garden, Chinese Academy of Sciences,** 723 Xinke Rd. Tianhe District, Guangzhou 510650, PR China

**^2^ College of Life Science,** University of Chinese Academy of Sciences, 19A Yuquan Road, Shijingshan District, Beijing 100049, PR China

**^3^  Department of Soil Science of Temperate Ecosystems, University of Göttingen, Büsgenweg 2, 37077 Göttingen, Germany**

*Correspondence:
Dr. Weijun Shen

Email: shenweij@scbg.ac.cn

Tel.: + 86 20 3725 2950

Fax: + 86 20 3725 2950

- 1. **Supplementary Figures and Table**

**1.1 Supplementary Figures**

**
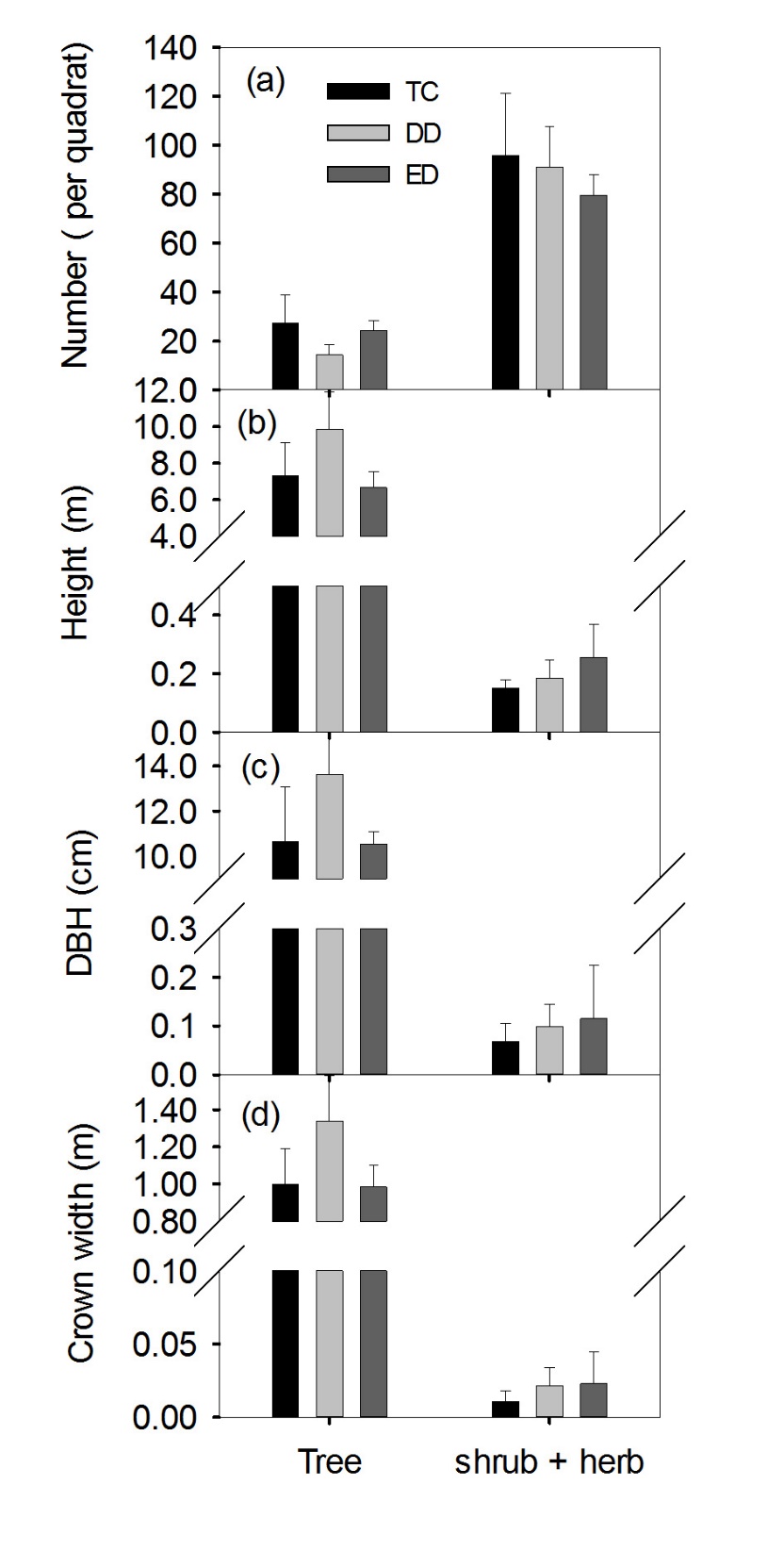
**

**Figure S1** General information about the vegetation features in control, drier dry season and wetter wet season (DD) and extended dry season and wetter wet season (ED) plots.




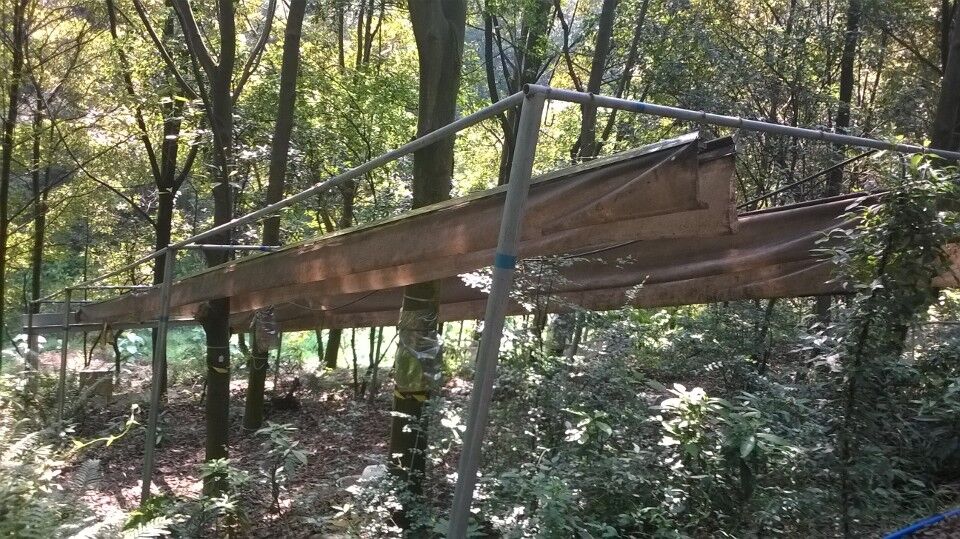


**Figure S2** Layout of the precipitation manipulating facilities including supporting structures, rainout shelters and water addition subsystems.

**
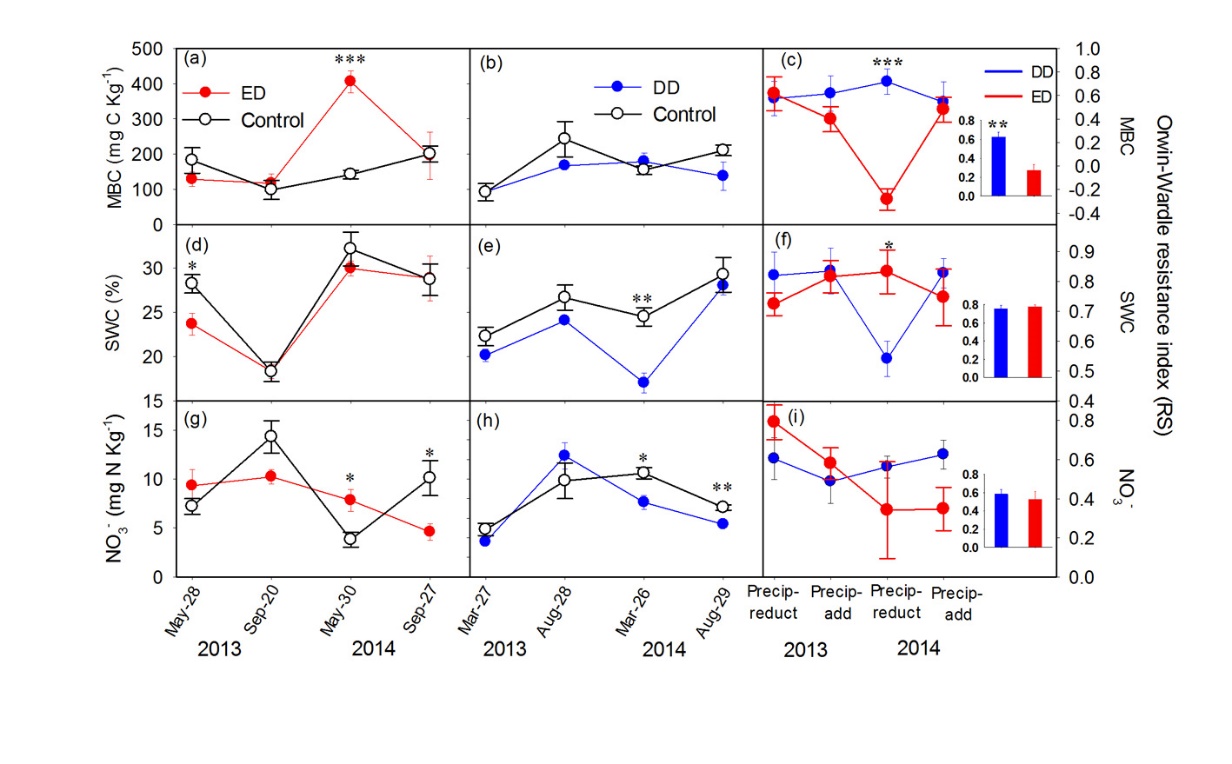
**

**Figure S3** Dynamics of (a-c) MBC, (d-f) SWC, and (g-i) NO_3_^-^ concentration in control and treatment plots and their resistance indices over the course of experiment (n = 4). The insert bar charts represent the average values of resistance index in each treatment during the experiment. The y-axis of the middle and left panels is presented on the left side, and the y-axis of the right panel is presented on the right side. The significance of the difference between treatment and control at each sampling point is presented as: *** *p* < 0.001, ** *p* < 0.01, * *p* < 0.05. Abbreviations: MBC, microbial biomass carbon; SWC, soil water content; DD, dryer dry season and wetter wet season; ED, extending dry season and wetter late wet season; Precip-red, precipitation reduction; Precip-add, precipitation addition.

- 1. **Supplementary Tables**

**Table S1** The outline of our treatments: dryer dry season and wetter wet season (DD), extending dry season and wetter wet season (ED). The exact date and amount of precipitation reduction and addition within each treatment were displayed.

| Treatment | DD | | | | |  | ED | | | | |
| --- | --- | --- | --- | --- | --- | --- | --- | --- | --- | --- | --- |
|  | Normal  precipitation | Throughfall reduction (drought) | | Rainwater addition  (storm) | |  | Normal  precipitation | Throughfall reduction (drought) | | Rainwater addition  (storm) | |
| 2013 | 506mm | From  01-October-2012  To  31-March-2013 | 220mm | 20-June-2013 | 55mm |  | 544mm | From  01-April-2013  To  31-May-2013 | 183mm | 28-June-2013 | 46mm |
|  |  |  |  | 19-July-2013 | 55mm |  |  |  |  | 19-July-2013 | 46mm |
|  |  |  |  | 22-August-2013 | 55mm |  |  |  |  | 31-July-2013 | 46mm |
|  |  |  |  | 15-September | 55mm |  |  |  |  | 31-August-2013 | 46mm |
|  |  |  |  |  |  |  |  |  |  | 20-September-2013 | 46mm |
| 2014 | 382mm | From  01-October-2013  To  31-March-2014 | 171mm | 19-June-2013 | 57mm |  | 545mm | From  01-April-2014  To  31-May-2014 | 270  mm | 29-July-2014 | 54mm |
|  |  |  |  | 20-July-2013 | 57mm |  |  |  |  | 30-August-2014 | 54mm |
|  |  |  |  | 21-August-2013 | 57mm |  |  |  |  | 23-September-2014 | 54mm |
|  |  |  |  |  |  |  |  |  |  | 24-September-2014 | 54mm |
|  |  |  |  |  |  |  |  |  |  | 27-September-2014 | 54mm |
|  |  |  |  |  |  |  |  |  |  |  |  |

**Table S2** The baseline values of soil physicochemical properties in treatment and control plots (means and standard error, *n* = 4) prior to experiments from May to September, 2012. Differences between plots are assessed by One-way ANOVA, and indicated by different lowercases. Abbreviates: DD, dryer dry season and wetter wet season; ED, extending dry season and wetter late wet season; SWC, soil water content; NO_3_^-^ and NH_4_^+^ contents (mg kg^-1^); MBC, microbial biomass carbon (mg kg^-1^); TN, total nitrogen; TP, total phosphorus; TOC, total organic carbon (mg kg^-1^); NNR, net nitrification rate (mg N kg^-1^ mon^-1^); NMR, net N mineralization rate (mg N kg^-1^ mon^-1^).

| Sampling time | plot | SWC (%) | NO_3_^-^ | NH_4_^+^ | MBC | TN (%) | TP (%) | TOC (%) | NNR | NMR |
| --- | --- | --- | --- | --- | --- | --- | --- | --- | --- | --- |
| 27-May-12 | Control | 28.4(1.0) | 2.7(0.6) | 4.0(0.1) | 77.1(10.2) | 0.23(0.02) | 0.022(0.002) | 3.5(0.4) |  |  |
|  | DD | 29.5(1.2) | 2.8(0.3) | 3.4(0.6) | 136.9(14.8) | 0.21(0.01) | 0.022(0.002) | 3.2(0.2) |  |  |
|  | ED | 27.4(1.1) | 2.3 (0.7) | 4.7(0.5) | 123.9(14.8) | 0.23(0.01) | 0.022(0.001) | 3.4(0.2) |  |  |
| 27-Jun-12 | Control | 22.4(0.9) | 1.6(0.3) | 5.6(1.2) | 166.3(9.6) | 0.17(0.01) | 0.023(0.002) |  | 7.2(0.8)a | 13.2(1.2) |
|  | DD | 24.7(0.9) | 1.7(0.4) | 5.8(1.0) | 140.8(13.5) | 0.14(0.01) | 0.020(0.002) |  | 4.9(1.3)ab | 10.5(1.5 |
|  | ED | 27.0(1.2) | 0.8(0.7) | 10.7(2.9) | 143.0(22.1) | 0.17(0.01) | 0.021(0.001) |  | 3.0(0.8)b | 14.4(2.1) |
| 15-Aug-12 | Control | 24.3(1.5) | 4.8(0.4) | 2.5(0.6) | 215.3(14.0) | 0.20(0.01) | 0.026(0.002) | 3.4(0.3) |  |  |
|  | DD | 26.4(0.7) | 3.0(0.7) | 3.4(0.8) | 152.8(6.0) | 0.14(0.02) | 0.021(0.002) | 2.7(0.2) |  |  |
|  | ED | 28.5(0.7) | 3.9(0.9) | 4.8(0.5) | 223.9(51.5) | 0.19(0.02) | 0.022(0.001) | 3.4(0.4) |  |  |
| 16-Sep-12 | Control | 24.5(1.1) | 2.9(0.5) | 3.3(1.0) | 190.9(31.0) | |  |  | 0.9(0.9) | 4.9(2.6) |
|  | DD | 22.2(0.9) | 5.7(1.3) | 1.4(0.3) | 230.9(38.6) | |  |  | 5.8(1.4) | 6.3(2.2) |
|  | ED | 21.8(1.3) | 3.0(1.1) | 5.6(2.0) | 216.7(11.8) | |  |  | 1.5(0.7) | 4.6(1.5) |
| Mean | Control | 25.0(1.0) | 3.0(0.1) | 3.8(0.6) | 162.4(6.4) | 0.20(0.01) | 0.024(0.002) | 3.5(0.3) | 4.1(0.8) | 9.0(1.9) |
|  | DD | 26.9(0.8) | 3.3(0.3) | 3.5(0.4) | 165.3(17.4) | 0.16(0.01) | 0.021(0.002) | 3.0(0.2) | 5.3(1.2) | 8.4(1.2) |
|  | ED | 26.9(0.8) | 2.5(0.8) | 6.4(1.2) | 176.9(17.4) | 0.19(0.01) | 0.022(0.001) | 3.4(0.3) | 2.3(0.7) | 9.5(1.2) |
